# Angioscopic and optical coherence tomographic evaluation of neointimal coverage: 9 months after expandable polyterafluoroethylene covered stent implantation

**DOI:** 10.1007/s00380-017-0964-9

**Published:** 2017-03-13

**Authors:** Ken Kongoji, Yuki Ishibashi, Nozomi Kotoku, Mizuho Kasahara, Hiroshi Yamazaki, Takanobu Mitarai, Ryo Kamijima, Kohei Koyama, Kihei Yoneyama, Yasuhiro Tanabe, Yoshihiro J. Akashi

**Affiliations:** 1 Division of Cardiology, Department of Internal Medicine, Kyorin University School of Medicine, 6-20-2, Shinkawa, Mitaka City, Tokyo 181-8611 Japan; 2 Division of Cardiology, Department of Internal Medicine, St. Marianna University School of Medicine, Kawasaki, Japan; 3 Division of Cardiology, Department of Internal Medicine, St. Marianna University School of Medicine, Yokohama City Seibu Hospital, Yokohama, Japan

**Keywords:** ePTFE covered stent, Angioscopy, Neointimal coverage

## Abstract

An expandable polytetrafluoroethylene (ePTFE) covered stent is generally employed to seal coronary artery perforation. The frequency of ePTFE covered stent use is relatively low; thus, only a handful of studies have reported neointimal coverage and endothelialization inside the deployed ePTFE and clinical time course after ePTFE implantation. This case report presents a 78-year-old man treated with an ePTFE covered stent when he suffered from coronary artery perforation after the implantation of two everolimus eluting stents in the left anterior descending artery. Follow-up coronary angiography 9 months after ePTFE covered stent implantation depicted favorable stent patency. Optical coherence tomography showed thin and uneven stent strut coverage at the culprit. Angioscopy also depicted partial white-coated coverage and stent strut exposure. The outcome of this case suggested that long-term dual antiplatelet therapy should be prescribed for preventing thrombosis after ePTFE covered stent implantation.

## Background

An expandable polytetrafluoroethylene (ePTFE) covered stent (GRAFTMASTER RX, Abbott Vascular Instruments, Abbott Park, IN, USA) is a sealing stent employed for coronary artery perforation [1] and saphenous vein graft lesions [2, 3]. Since an ePTFE covered stent is mainly used in the emergency cases, its frequency is relative low. Neointimal coverage and endothelialization after ePTFE covered stent implantation or its clinical time course has not been fully reported. Here, we report a case with coronary artery perforation treated with an ePTFE covered stent and followed up using coronary angiography, optical coherence tomography (OCT) and angioscopy 9 months after implantation.

## Case report

**Fig. 1:**
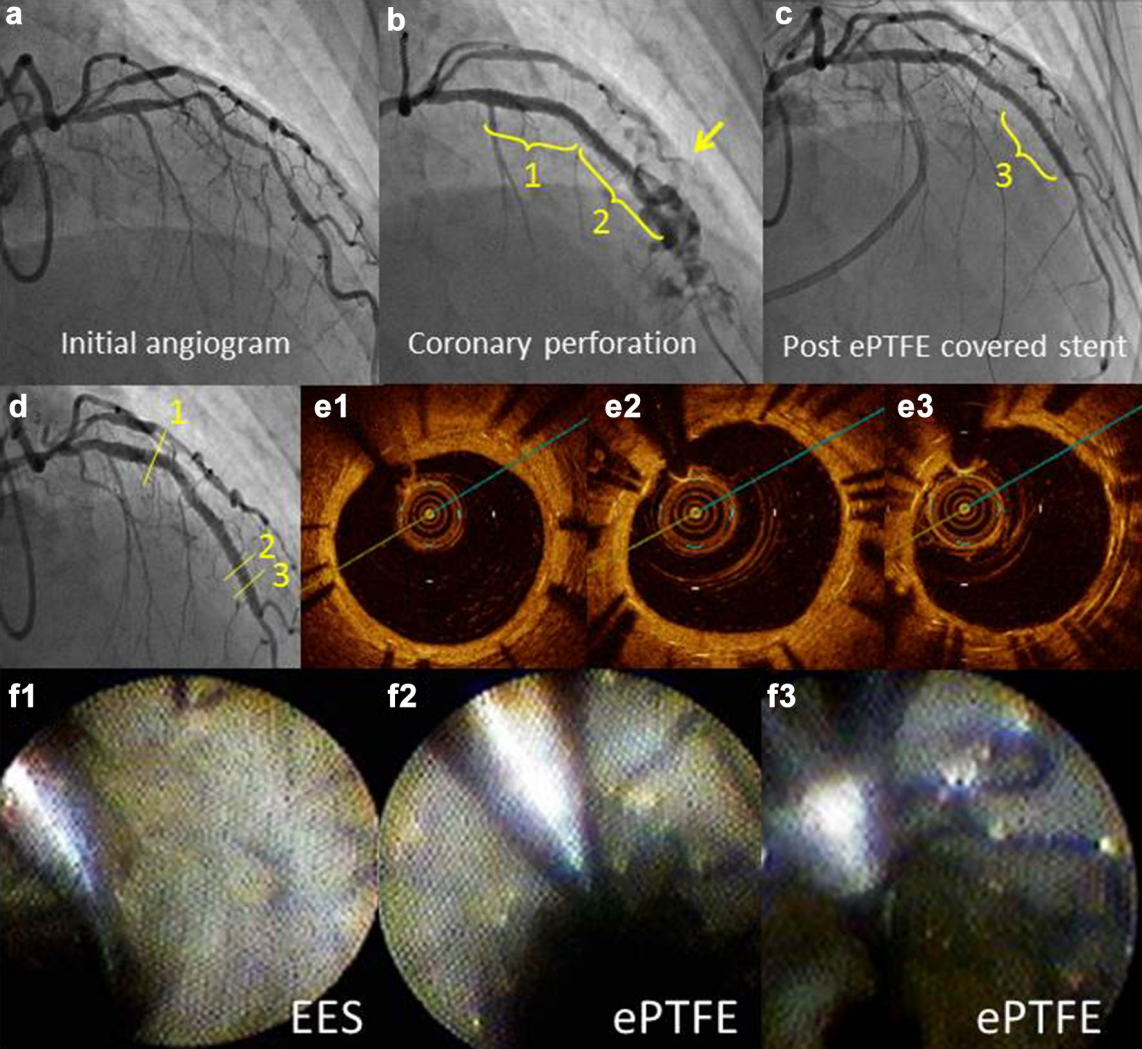
**a** Severe stenosis was depicted at the left anterior dissenting artery. **b** Coronary perforation after stent implantation (*yellow arrow*). *1, 2* Everolimus eluting stent (EES). **c** Post expandable polytetrafluoroethylene (ePTFE) covered stent implantation (*3*). **d** Coronary angiograms 9 months after stent implantation. **e1** Optical coherence tomographic images at the middle of proximal EES. The EES struts are completely covered by neointima. At the middle of ePTFE stent. The double and triple stent struts can be observed. (**e2**) depicts uneven and partial coverage, whereas (**e3**) shows no circumferential coverage. Angioscopic images of ePTFE covered stent and EES struts. **f1** The EES is completely covered by white neointima; however, the strut is translucently identified. **f2** Partial coverage with white tissue in the ePTFE covered stent. **f3** ePTFE stent strut exposure

The patient was a 78-year-old man, who previously suffered from acute myocardial infarction and received a bare metal stent implantation at the right coronary artery in January, 2013. Since coronary angiography showed a moderate stenosis in the left anterior descending artery (LAD), he underwent stress myocardial perfusion scintigraphy with thallium, resulting in redistribution in the anterior septal wall area. In August, 2013, percutaneous coronary intervention (PCI) was performed at the culprit lesion. The culprit lesion was tortuous and angulated. Moreover vessel size tapered distally (Fig. 1a). Two EESs (everolimus eluting stents; Xience expedition 3.0 × 33 mm, 3.5 × 18 mm, Abbott Vascular Instruments, Abbott Park, IN, USA) were successfully implanted to cover the lesion in the LAD and distal stent area including the overlapping segment of EESs was additionally dilated with a 3.5-mm stent delivery balloon. However, after balloon inflation, angiography confirmed the presence of Type III coronary perforation (contrast streaming or cavity spilling) at distal stent site (Fig. 1b). The perfusion balloon was inflated for 30 min, however, coronary angiography showed residual Type II perforation. Therefore, immediately, the area of perforation was sealed with the use of an ePTFE covered stent 3.0 × 16 mm (Fig. 1c). The patient was discharged on the 12th hospital day and antiplatelet drugs (aspirin 100-mg and 75-mg clopidogrel, daily) were continuously prescribed. Follow-up coronary angiography 9 months after ePTFE covered stent implantation depicted favorable stent patency (Fig. 1d). Thin and uneven stent strut coverage by neointimal coverage in the single EES strut was detected by optical coherence tomography (OCT), while partial tissue coverage in the ePTFE covered stent strut was detected (Fig. 1e1–3). The images obtained by angioscopy were similar to those of OCT; the ePTFE covered stent strut was partially coated by white tissue or exposed. No obvious silent stent thrombus was found (Fig. 1f1–3). In this case, the patient had an uneventful clinical course beyond 3 years.

## Discussion

In this case, OCT and angioscopy depicted the thinly coated ePTFE covered stent strut with white tissue. It is quite challenging to distinguish this coverage, neointima or organized thrombus. The surface of fresh fibrin thrombus is generally not smooth [4]. In this case, OCT depicted the smooth surface, suggesting that it might be neointimal coverage or organized thrombus. Moreover, the absence of obvious white or red thrombi in the ePTFE covered stent supported our theory. The neointimal coverage was observed in the most part of the overlapping EES struts, whereas partial and thin coverage or strut exposure was found in the ePTFE stent strut. An ePTFE covered stent might be delayed the neointimal coverage compared to EES because it might inhibit smooth muscle cell migration in the stent strut. According to previous report, thromboembolism might possibly occur 9 months after ePTFE covered stent implantation. Therefore, the long-term therapy with antiplatelet drugs or anticoagulants is thus required in such cases. RECOVERS trial [3] was a randomized and multicenter trial to evaluate the usefulness of an ePTFE covered stent compared with a bare metal stent in the saphenous vein graft lesions. No differences in binary stenosis or target lesion revascularization were found between ePTFE covered stents and bare metal stents, although, the 6-month non-Q-wave myocardial infarction rate was significantly higher in the lesions treated with ePTFE covered stents. Moreover, it reported the occurrence of subacute thrombosis in two patients treated with ePTFE covered stents. In the RECOVERS trial, dual antiplatelet therapy (DAPT) was mandatory to take only for 3 months; then, the single use of aspirin was continuously prescribed after the termination of DAPT. Considering uneventful clinical course beyond 3 years in this case, it may be recommended to avoid stent thrombosis after artificial covered stent deployment. Given the paucity of data, the optimal duration of DAPT after implantation of ePTFE covered stent should be assessed in future studies.

## Conclusion

This is the rare case report observed using coronary imaging 9 months after ePTFE covered stent implantation. Although the type and optimal duration of dual antiplatelet therapy (DAPT) for patients undergoing an ePTFE implant has not been studied, longer DAPT should be recommended for preventing stent thrombosis after ePTFE covered stent implantation.
